# Evaluation of fixed-panel, multicolour ClearLLab 10C at an academic flow cytometry laboratory in Johannesburg, South Africa

**DOI:** 10.4102/ajlm.v11i1.1458

**Published:** 2022-07-15

**Authors:** Deborah K. Glencross, Leanne Swart, Melanie Pretorius, Denise Lawrie

**Affiliations:** 1 Department of Molecular Medicine and Haematology, Faculty of Health Sciences, University of the Witwatersrand, Johannesburg, South Africa; 2 Department of Molecular Medicine and Haematology, Charlotte Maxeke Academic Hospital, National Health Laboratory Service, Johannesburg, South Africa

**Keywords:** ClearLLab 10C, immunophenotyping, fixed-panel, standardisation, multicolour, leukaemia, lymphoma, diagnostics

## Abstract

**Background:**

Flow cytometric immunophenotyping is well established for the diagnosis of haematological neoplasms. New commercially available systems offer fixed, pre-aliquoted multi-parameter analysis to simplify sample preparation and standardise data analysis.

**Objective:**

The Beckman Coulter (BC) ClearLLab™ 10C (4-tube) system was evaluated against an existing laboratory developed test (LDT).

**Methods:**

Peripheral blood and bone marrow aspirates (*n* = 101), tested between August 2019 and November 2019 at an academic pathology laboratory in Johannesburg, South Africa, were analysed. Following daily instrument quality control, samples were prepared for LDT (using > 20 2–4-colour in-house panels and an extensive liquid monoclonal reagent repertoire) or ClearLLab 10C, and respectively analysed using in-house protocols on a Becton Dickinson FACSCalibur, or manufacturer-directed protocols on a BC Navios. Becton Dickinson Paint-a-Gate or BC Kaluza C software facilitated data interpretation. Diagnostic accuracy (concordance) was established by calculating sensitivity and specificity outcomes.

**Results:**

Excellent agreement (clinical diagnostic concordance) with 100% specificity and sensitivity was established between LDT and ClearLLab 10C in 67 patients with a haematological neoplasm and 34 participants with no haematological disease. Similar acceptable diagnostic concordance (97%) was noted when comparing ClearLLab 10C to clinicopathological outcomes. Additionally, the ClearLLab 10C panels, analysed with Kaluza C software, enabled simultaneous discrimination of disease and concurrent background myeloid and lymphoid haematological populations, including assessing stages of maturation or sub-populations.

**Conclusion:**

ClearLLab 10C panels provide excellent agreement to existing LDTs and may reliably be used for immunophenotyping of haematological neoplasms, simplifying and standardising sample preparation and data acquisition.

## Introduction

Flow cytometric immunophenotyping is an established methodology for the diagnosis of haematological neoplasms^1,2,3,4,5,6^ outlined in the World Health Organization classification of haemopoietic and lymphoid tissues.^7,8^ Published protocols for sophisticated multicolour flow cytometry systems have markedly expanded the scope of routine immunophenotypic testing and improved diagnostic capabilities, allowing for simultaneous multi-parameter examination and documentation of specific neoplastic disease immunophenotypes alongside normal haematological development.^9,10,11,12,13,14^ Rare event populations, however small, are likewise identified to reveal evidence of minimal residual disease.^13,15,16^ In addition, harmonised approaches, including standardised multi-parameter marker staining, instrument setup and data collection, and automated gating, can reduce test variability, streamline analysis for immunophenotyping and ensure reproducible outcomes between and within centres.^17,18^

Leading experienced and knowledgeable groups have published specific panels and protocols for multi-parametric flow cytometry immunophenotyping method setups, panel selection and data analysis,^9,19,20^ irrespective of the flow cytometer used. However, multi-parametric flow cytometry is not straightforward^18,19,20,21,22,23,24^; the setup is complex, requiring expertise in flow cytometry techniques.^20,25^ Therefore, many testing facilities opt to retain their in-house-developed predicate testing methods to meet their local service needs.^26^

To meet these challenges, Beckman Coulter (BC) launched a United States Food and Drug Administration-approved, standardised, fixed multicolour ClearLLab reagent system for leukaemia and lymphoma immunophenotyping in 2019.^27,28^ The four 10-colour marker panels facilitate the detection of most acute leukaemias and lymphomas. When used with a BC Navios instrument, the system offers the advantages of multi-parametric analysis but with simplified pre-prepared marker panels and standardised instrument setup and quality control.^28^ This study aimed to evaluate the ClearLLab™ 10C system (including the B-cell, T-cell, M1 and M2 diagnostic tubes)^27,28^ against our existing predicate method (utilising > 20 in-house, 2–4-colour fluorescence panels and a vast liquid monoclonal reagent repertoire) in our busy, academic flow cytometry laboratory. The laboratory offers leukaemia and lymphoma immunophenotyping services at the National Health Laboratory Service, Johannesburg, South Africa. A secondary aim of this study was to compare the ClearLLab 10C system against other routinely employed clinicopathological diagnostic methods used in our site for detecting haematological neoplasms, including morphological and/ or histological review.

## Methods

### Ethical considerations

Ethics clearance for this study was obtained from the University of the Witwatersrand Ethics Committee (HREC) (M1704129). The HREC waived patient consent as validation for ClearLLab 10C testing utilised remnant samples previously submitted for routine diagnostic testing. All flow cytometric data analysed were de-identified; data were therefore effectively anonymised, and no patient identifiers were used.

### Study design and site

This prospective observational cohort study^29^ was performed at a national referral flow cytometry laboratory at the Charlotte Maxeke Johannesburg Academic National Health Laboratory Service Flow Cytometry Laboratory, Johannesburg, South Africa. The unit is an academic testing facility with a 300-sample monthly workload, referred from four large sister academic hospitals, other district-level facilities, and other regional centres across the national network. In addition, the laboratory participates in the United Kingdom National External Quality Assessment Scheme (Sheffield, United Kingdom) proficiency testing scheme for leukaemia immunophenotyping (Part 1) and Leukaemia Diagnostic Interpretation (Part 2).^30^

### Specimens

Bodily specimens, including bone marrow aspirates, peripheral blood, cerebrospinal and pleural fluid, and lymph node tissue, referred to the National Health Laboratory Service in Johannesburg, South Africa, for leukaemia immunophenotyping by local sister hospitals were included in the study. After diagnostic testing with the laboratory developed test (LDT) was completed, the same sample was tested using the index method (ClearLLab 10C), provided that sufficient prepared cell concentrate for at least 4 aliquots of concentrated sample, or at least 1 mL of the whole sample, remained. All specimens for ClearLLab 10C evaluation were selected per the Bethesda guidelines.^2^ The guideline explicitly outlines the appropriate use of flow cytometry for patients with clinically suspected oncological haematolymphoid neoplasms or patients with previously diagnosed oncological haematolymphoid disease enrolled for treatment. Samples with no pertinent history or clinical suspicion of haematological disease, older than 24 h, visibly haemolysed or clotted, with insufficient volume at receipt of the sample, or insufficient remnant sample to undertake index testing, were excluded from the study. One hundred and one remnant specimens (from 36 children and 65 adults from any ethnic or racial background) were processed for ClearLLab 10C from August through November 2019: 82 bone marrow aspirate samples and 15 peripheral blood samples were collected into dipotassium ethylenediaminetetraacetic acid. Three bodily fluid samples (two cerebrospinal and one pleural fluid) were submitted; a single lymph node biopsy was collected into saline.

### Sample preparation and cell concentrates

For this study, the existing predicate method is the LDT; samples were analysed on the Becton Dickinson (BD) FACSCalibur (San Hose, California, United States). The index method is the ClearLLab 10C panel; samples were analysed on a Beckman Navios flow cytometer (BC, Miami, Florida, United States).

All sample cell concentrates were prepared for flow cytometric analysis according to current local standard operating procedures, irrespective of reagents used. Two to four 500 µL aliquots of blood or bone marrow per patient were prepared depending on the initial white blood cell count. The aliquots of the sample were placed in conical centrifuge tubes to which 14.5 mL of 0.899% solution of ammonium chloride (NH_4_CL) containing 0.084% sodium bicarbonate and 0.0037% ethylenediaminetetraacetic acid (Merck, Darmstadt, Germany) was added to lyse the red blood cells. This was allowed to stand for 15 min, followed by a short 3-min spin at 3000 rpm. A further 5-min incubation time in the NH_4_CL solution, followed by a 3-min spin, was performed if the red cells were macroscopically visible on the cell pellet. Samples were subsequently washed three times with a commercial phosphate-buffered saline (PBS) solution at pH 7.3 ± 2 (Oxoid LTD, Basingstoke, United Kingdom) containing 0.09% sodium azide (Merck, Darmstadt, Germany) and 0.2% bovine serum albumin (Biowest, Nuaille, France). Cell pellets were resuspended with 1 mL PBS. White cell count was obtained for each sample using 100 µL of the cell concentrate and 100 µL of BC FlowCount™^31,32^ beads (BC, Miami, Florida, United States) in a 1 mL PBS solution on the Navios flow cytometer. The volume of cell concentrate estimated to contain ~1 × 10^6^ cells per aliquot was calculated, and this predetermined cell concentrate volume was added to each labelled marker tube of the LDT panel first. Remnant cell concentrates, or cell concentrates harvested from whole remnant samples, were aliquoted into the ClearLLab tubes. Each sample aliquot was incubated in the dark at room temperature (22 °C) for 15 min, followed by a final wash in PBS. The cell pellet was reconstituted with 700 µL of PBS and immediately run on either the FACSCalibur flow cytometer (BD Biosciences, San Jose, California, United States) for the LDT or the Navios flow cytometer (BC, Miami, Florida, United States) for the ClearLLab samples. Whole samples (remaining after cell concentrate preparation) were stored on the bench at room temperature (~22 °C) during testing; after analysis was completed, remnants of both the whole sample and the prepared samples were refrigerated at 4 °C.

### Flow cytometer quality control and immunophenotyping

#### Laboratory developed test method

Before March 2020, the LDT used the BD FACSCalibur™ flow cytometer. Daily quality control for the LDT on the BD FACSCalibur included assessment of background contamination, carryover, acquisition and analysis of manufacturer-recommended 3-colour and APC Calibrite beads (BD Biosciences, San Jose, California, United States) and acquisition and analysis of Immunotrol^TM^ process control (BC Inc., Brea, California, United States), using four monoclonal antibodies namely CD45 (PerCP), CD3 (APC), CD14 (FITC) and CD13 (PE). Liquid monoclonals were used according to the manufacturer specifications and manually pipetted individually to constitute 2–4-colour assembled panels using varying combinations of cell markers chosen by attending pathologists according to the merits of the patient’s presenting case history (Table 1). The patients’ samples were acquired on BD FACSCalibur™ using CellQuest™ software (BD Biosciences, San Jose, California, United States); the instrument typically stops counting at 5000 events. In the event of a paucicellular sample, the tube would run for the maximum time permissible ~300 s, to ensure as many cells as possible could be counted, but consequently with total variable events counted. Listmode (.fcs) data files were analysed and interpreted by consultant hematopathologists using BD Paint-a-Gate™ software (BD Biosciences, San Jose, California, United States).

**TABLE 1:** Summary of the laboratory developed test panels and monoclonal antibody combinations and respective fluorochromes used during routine diagnostic workup at the Charlotte Maxeke Johannesburg Academic Laboratory in Johannesburg, South Africa, August 2019 – November 2019.

Variations of LDT marker combinations used	Monoclonal reagent	Manufacturer or supplier	Intended use
1. CD235/CD45 2. CD14/CD45	CD235a (FITC), also known as anti-glycophorin A CD45 (PE)	Beckman Coulter Marseille, France Becton Dickinson, San Jose, California	Screening in the majority of cases to identify red cell contamination after red cell lysis, together with pan-leucocyte marker used to discern various white blood cell populations and CD14 to reveal the proportion of mature monocytes
3. CD45 as the third colour in any four colour panel	CD45 (PerCP)	Becton Dickinson, San Jose, California	Used to define pan-leucocyte in a four colour analysis as shown below
4. CD2/CD5 5. CD4/CD8 or 6. CD4/CD8/CD3/CD45 7. CD34/CD7 8. CD1a† 9. Cytoplasmic CD3† 10. CD3/CD25† 11. CD30†	CD2 (FITC)	Becton Dickinson, San Jose, California	T-cell antigen discernment in TALL or lymphoblastic lymphoma or mature T-cell LPD
	CD3 (FITC)	Becton Dickinson, San Jose, California	
	CD4 (FITC)	Beckman Coulter Inc, Brea, California	
	CD5 (PE)	Becton Dickinson, San Jose, California	
	CD8 (PE)	Beckman Coulter Inc, Brea, California	
	CD7 (PE)	Beckman Coulter Inc, Brea, California	TALL or lymphoblastic lymph
	CD34 (FITC)	Dako-Agilent, Santa Clara, California	
	CD1a (PE)	Becton Dickinson, San Jose, California	CD1a used to discern TALL / LL
	cytoplasmic CD3 (FITC)	Becton Dickinson, San Jose, California	TALL
	CD25 (PE)†	Dako-Agilent, Santa Clara, California	CD25 Adult T-cell leukaemia/ lymphoma
	CD30 (FITC)	Beckman Coulter Inc, Brea, California	Anaplastic large cell lymphoma (T-cell)
12. Kappa/Lambda in combinations as below	Kappa (FITC) Lambda (PE)	Both Dako-Agilent, Santa Clara, California	Clonality in B-cell lymphoproliferative disorder
13. CD19/CD10 14. CD19/CD10/CD45/CD34 15. Kappa/LambdaCD19/CD10 16. Kap‡/Lam‡/CD19 CD34 17. CD19/-/CD45/CD34† 18. Cytoplasmic CD22† 19. Cytoplasmic CD79a† 20. Kappa/Lambda CD19/CD5 21. CD23/CD20 22. CD19/CD5/CD20/CD23 23. FMC7/CD10† 24. FMC7/CD10/CD45/CD22 25. CD103/CD25† 26. CD11c/CD25†	CD10 (PE)	Beckman Coulter Inc, Brea, California	B-cell ALL, or Burkitt Lymphoma, versus precursor B-cell haematogones
	or CD10 (APC)	Becton Dickinson Inc, San Jose, California	
	CD19 (FITC)	Becton Dickinson Inc, San Jose, California	
	or CD19 (PerCP-Cy5.5)	Becton Dickinson Inc, San Jose, California	
	CD34 (APC)	Becton Dickinson Inc, San Jose, California	
	Cytoplasmic CD22 (PE)	Dako-Agilent, Santa Clara, California	Precursor B-cell ALL
	Cytoplasmic CD79a (PE)	Becton Dickinson, San Jose, California	Precursor B-cell ALL
	CD20 (PE)	Beckman Coulter Inc, Brea, California	B-cell CLL
	or CD20 (PERCPCy5)	Becton Dickinson Inc, San Jose, California Inc, San Jose, California	
	CD23 (FITC)	Dako-Agilent Inc, Santa Clara, California	
	or CD23 (APC)	Becton Dickinson Inc, San Jose, California Inc, San Jose, California	
	FMC-7(FITC)	Beckman Coulter Marseille, France	Follicular lymphoma
	CD10 (PE)	Beckman Coulter Inc, Brea, California	
	CD22(APC)	Becton Dickinson, San Jose, California	
	CD103 (FITC)	Becton Dickinson, Marseille, France	Hairy cell leukaemia
	CD11c (FITC)	Dako-Agilent, Santa Clara, California	
	CD25 (PE)	Dako-Agilent, Santa Clara, California	
27. Nuclear Terminal deoxynucleotidyl transferase†	Nuclear TdT(FITC)	Dako-Agilent, Santa Clara, California	All B-cell and TALL
28. Cytoplasmic myeloperoxidase (MPO)† 29. CD11b/CD13 30. CD15/CD117 31. HLA-DR/CD33 32. CD64/-/CD45/CD34† 33. CD42a/CD61†	Cytoplasmic MPO (FITC)	Dako-Agilent, Santa Clara, California	Acute myeloid leukaemia
	CD11b (FITC)	Beckman Coulter Inc, Brea, California	Acute myeloid leukaemia
	CD13 (PE)	Beckman Coulter Inc, Brea, California	
	CD15 (FITC)	Dako-Agilent, Santa Clara, California	Acute myeloid leukaemia
	CD117 (PE)	Beckman Coulter, Marseille, France	
	HLA-DR (FITC CD33) (PE)	Both Becton Dickinson, San Jose, California	Acute myeloid leukaemia
	CD64 (FITC)	Beckman Coulter, Marseille, France	Acute myeloid leukaemia, brighter CD64 confirming a monocytic component
	CD42a (FITC) and CD61 (PE)	Both Dako-Agilent Santa Clara, California	Discern megakaryoblastic subtype in acute myeloid leukaemia
34. CD19/CD138/-/200† 35. CD38/CD56/CD45†	CD19 (FITC)	Becton Dickinson San Jose, California	Plasma cell dyscrasias
	CD138 (PE)	Beckman Coulter, Marseille, France	
	CD200 (APC)	Invitrogen-Fisher Scientific Inc, Pittsburgh, Philadelphia	
	CD38 (FITC)	Dako-Agilent, Santa Clara, California	
	CD56 (PE)	Beckman Coulter, Brea, California	
36. IgG1/IgG1 37. MsIgG2a/MsIgG2b 38. MsIgM/MsIgG1	All isotypic controls MsIgG 1(FITC) MsIgG 1 (PE) MsIgG 2b (FITC) MsIgG 2a (PE) MsIgM (FITC) MsIgG 1 (PE)	All Beckman Coulter Inc, Brea, California	Mouse (Ms) isotypes controls were previously included with all laboratory developed panels tested

**Note:** Total sample preparation and flow cytometric acquisition time: Laboratory developed test first-line analysis = Average 60–90 min per patient, excluding incubation and lyse steps but including adding of multiple cell aliquots and many monoclonal pipetting steps (greater than 40) followed by sample acquisition (from 1 to 5 min per tube); Laboratory developed test, optional second-line investigation† = Average 20–30 min of preparation and acquisition time per sample, excluding incubation of tubes.

Total preparation time per patient: 80–120 min.

All monoclonal markers noted are used for surface staining unless otherwise specified as either cytoplasmic or nuclear staining.

nm, nanometre; FITC, fluorescein isothiocyanate; PE, phycoerythrin; PerCP, peridinin chlorophyll; APC, allophycocyanin; MsIgG, mouse immunoglobulin G, type 1, 2a or 2b follows (isotypic control); MsIgM, mouse immunoglobulin type M (isotypic control); ALL, acute lymphoblastic leukaemia; CLL, chronic lymphocytic leukaemia; TALL, T-cell acute lymphoblastic leukaemia; LDT, laboratory developed test; LPD, lymphoproliferative disorder.

† , Includes cytoplasmic studies for acute myeloid or lymphoblastic leukaemia, further investigation of mature B-cell lymphoproliferative disorders or plasma cell dyscrasias;

‡ , Anti-kappa or anti-lambda light chain analysis to confirm clonality in B-cell lymphoproliferative disorders.

#### ClearLLab 10C panel method

Before starting the study, BC provided training for the Navios instrument, including application setup, control runs and panel acquisition. Colour compensation for ClearLLab testing was performed according to manufacturer specifications.^28^ Before commencing the validation study, an initial reproducibility study was performed (data not shown), utilising both the normal and abnormal controls (10 for each of the respective ClearLLab tubes; see Table 2 for details of markers included in the ClearLLab tubes). The assay values obtained were within the published expected ranges of the package inserts; the reproducibility of the process controls met the manufacturer’s percentage coefficient of variation (%CV) of ≤ 10% for all targeted populations and repeatability of ≤ 20%CV for all targeted populations.^33^ Daily internal quality control for the Navios flow cytometer included a daily background count (with locally established target values of < 100 events/100 s) and daily carryover (locally established target value < 1% carryover). Daily Flow Check Pro (target HPCV < 2.0% for FL1-FL5, < 3% for FL7-FL8 and < 4% for FL9-FL10) was used to verify optical alignment and fluidics (BC, Lismeehan, Ireland) on the Navios, as well as standardise the optical and fluorescence settings (Flow-Set Pro, BC, Lismeehan, Ireland). ClearLLab ‘normal’ and ‘abnormal’ process control cells (BC, Lismeehan, Ireland) were used to verify sample processing, acquisition and analysis against established package insert values and assess repeatability over time (data not shown). Weekly reproducibility was ensured through locally established target values for the bead count rate^31,32^ and cell counts.^33^ The sensitivity and specificity of the ClearLLab were expected to match or exceed the published BC ClearLLab 10C acceptance limits of > 70% sensitivity and > 80%, specificity.

**TABLE 2:** Reagents used with the ClearLLab 10C panels verified at the Charlotte Maxeke Johannesburg Academic Laboratory in Johannesburg, South Africa, August 2019 – November 2019.

ClearLLab tube	Fluorochromes									
	Blue laser (488 nm excitation)					Red laser (638 nm)			Violet laser (405 nm)	
	FITC	PE	ECD	PC5.5	PC7	APC	APC A-700	APC A-750	PB	KrO
B-cell†	Kappa	Lambda	CD10‡	CD5‡	CD200	CD34	CD38‡	CD20	CD19	CD45
T-cell†	TCRγδ	CD4	CD2	CD56	CD5‡	CD34	CD7‡	CD8	CD3	CD45
M1-cell†	CD16	CD7‡	CD10‡	CD13‡	CD64	CD34	CD14	HLA-Dr‡	CD11b	CD45
M2-cell†	CD15	CD123	CD117	CD13‡	CD33	CD34	CD38‡	HLA-Dr‡	CD19	CD45

**Note:** Total sample preparation and flow cytometric acquisition time: ClearLLab 10C first-line analysis = Less than 1 min, including four cell aliquot pipetting steps (<1 min), ~2-min average sample acquisition per tube (8–10 min total); Laboratory developed test optional second-line investigation§ = Average 20–30 min of preparation and acquisition time per sample, excluding incubation of tubes.

Total max preparation per patient = 33–40 min.

B-cell lymphoproliferative disorders or plasma cell dyscrasias (see also Table 1 above).

nm, nanometre; FITC, fluorescein isothiocyanate; PE, phycoerythrin; ECD, energy coupled dye; PC5.5, phycoerythrin-conjugated cyanin 5.5; PC7, phycoerythrin-conjugated cyanin 7; APC, allophycocyanin; APC A-700, allophycocyanin-conjugated Alexa fluor 700; APC A-750, allophycocyanin-conjugated Alexa fluor 750; PB, pacific blue; KrO, krome orange.

† , All panels are available from Beckman Coulter, Miami, Florida.

‡ , Internal monoclonal reproducibility quality control is highlighted (i.e. repeated monoclonal reagents useful for confirming true marker expression and discerning possible spectral spill-over from bright antigen expression that may lead to over calling or false interpretation of expression).

§ , Includes cytoplasmic studies for acute myeloid or lymphoblastic leukaemia, further investigation of mature cells.

Samples were prepared according to manufacturer specifications but with a modification; the IOTest3 lysing solution was replaced with an NH_4_Cl solution. Following red cell lysis and wash steps, prepared ~10^6^ cell aliquots were added to each of the ClearLLab™ 10C B, T, M1 and M2 tubes (Table 2). After a 15-min incubation in the dark, tubes were again washed. All samples were acquired on a BC Navios™ Flow cytometer. At minimum, 50 000 to 100 000 events were acquired on all samples. Data files were analysed offline by trained attending hematopathologists using BC Kaluza C™ software (BC, Miami, Florida, United States).

### Assessment of immunophenotype

Immunophenotypes were established using appropriately applied, pathologist-defined, sub-population gates that were unique to each case analysed. For the LDT data, a combination of light scatter and CD45 or CD19 gating was used to identify target cell (neoplastic) populations. For the ClearLLab 10C, initial gating included isolation of singlets followed by primary gating on CD45 positive events, with secondary gating applied to the identified target population. The specific antigen expression was documented for both the haematological neoplasm present as well as the normal haematological populations in the background (as an internal control of marker expression), based on the expression of all the available markers (antigens) across the four panels included in the ClearLLab 10C panel set. The specific presence or absence of antigens, and the respective specific intensity of staining of each antigen, was used to establish the diagnosis of specific subtypes of leukaemia or lymphoma per the World Health Organization’s classification of haemopoietic and lymphoid tissues^7,8^. Also, the clinical history, other laboratory parameters (e.g. full blood count and white blood cells differential counts), and bone marrow aspirate morphology were taken into account.

### Statistical analysis

Data were collected and collated into Microsoft Excel (Redmond, Washington, United States) spreadsheets for data analysis. Sensitivity outcomes were calculated; the clinical accuracy of the qualitative flow cytometric results (concordance of diagnosis as normal or abnormal) between the LDT and ClearLLab 10C outcomes were assessed for samples tested, and a contingency sensitivity (as the percentage of patients with malignancy) and specificity (the percentage of patients with no malignancy) table was constructed. In addition, the positive predictive value, as the probability of malignant outcomes with an abnormal immunophenotype detected, as well as negative predictive value as the probability of non-malignant outcomes in a patient with no disease, were also calculated. Thereafter, the ClearLLab results of each patient were compared to their clinicopathological or haematological diagnosis based on a similar approach by Hedley et al.^28^ to demonstrate the objectivity of a comprehensive ClearLLab 10C diagnostic approach. Clinical concordance was achieved using the categories of ‘haematologically malignant’ or ‘haematologically non-malignant’, as suggested by the presence or absence of an abnormal immunophenotype. In this comparative analysis, both false positives and negatives, and true positives and negatives, as well as both positive predictive value and negative predictive value outcomes, were reported using ClearLLab outcomes as the objective standard.

## Results

### Daily quality control

The daily quality control results over the study period confirmed that daily background counts were consistently < 100 events in 100 s, carryover < 1% and Flow Check Pro results acceptable with HPCV < 2 for FL1 to FL5, HPCV < 3 for FL6 to FL8 and < 4 for FL9 and FL10 for the days that the patient samples were tested; Flow-Set Pro also confirmed acceptable fluorescence stability, with %CVs < 5% for the blue and violet laser (FL1–FL5 and FL9–FL10) and < 8% for the red laser (FL6–FL8). Further weekly CD4 reproducibility studies on the Navios confirmed that the flow count rate and absolute counts had a %CV < 5% for the duration of the validation. Precision and accuracy of ClearLLab controls were in agreement with the manufacturer’s claims for repeatability and accuracy of ClearLLab control cells (i.e. control cells assay values fell within the package insert limits and %CVs were consistently < 10%).

### Comparing ClearLLab 10C against predicate laboratory developed test

**FIGURE 1:**
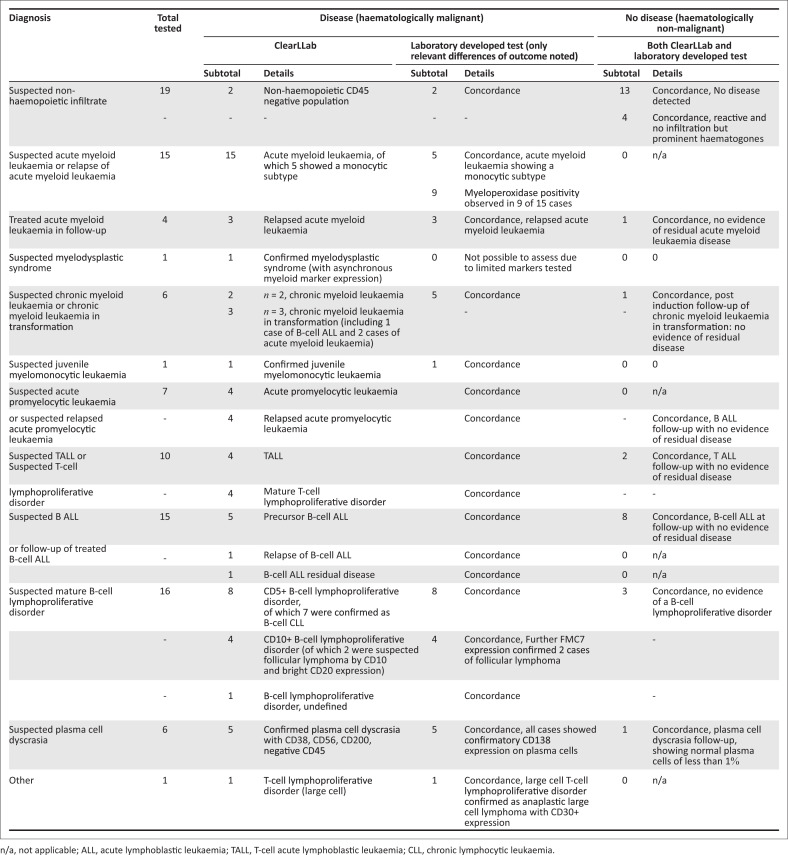
Comparison of diagnostic immunophenotypic outcomes of cases tested by laboratory developed test and ClearLLab 10C at the Charlotte Maxeke Johannesburg Academic Laboratory in Johannesburg, South Africa, August 2019 – November 2019.

**FIGURE 2:**
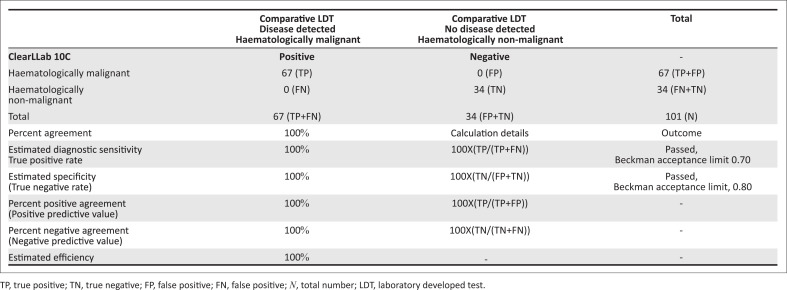
Part I: Comparison of diagnostic immunophenotypic outcomes of cases tested by the predicate laboratory developed test and ClearLLab 10C at the Charlotte Maxeke Johannesburg Academic Laboratory in Johannesburg, South Africa, during the period from August 2019 – November 2019.

One hundred and one bodily specimens from 36 children and 65 adults, irrespective of ethnic or racial background, were enrolled into the study. These included: 82 bone marrow aspirates, 15 peripheral blood samples, one pleural and two cerebrospinal fluids, and one lymph node biopsy. Sixty seven of the 101 cases tested had haematological malignancy, while 34 cases had no evidence of malignancy despite a clinical suspicion (Figure 1). Among three bodily fluid samples tested, two cerebrospinal fluid samples showed evidence of tumour infiltration, one with T-cell acute lymphoblastic leukaemia, and another infiltrated with acute myeloid leukaemia; a separate (third) pleural fluid sample showed infiltration by follicular lymphoma, confirmed with CD10 expression and other markers suggesting B-cell maturity. The lymph node biopsy had a large T-cell lymphoproliferative disorder; additional LDT testing revealed an anaplastic large cell lymphoma (confirmed with CD30 expression). A diagnostic concordance of 100% was noted between the ClearLLab outcomes and the LDT, revealing identical diagnostic outcomes (Figure 2). These findings exceeded the manufacturer acceptance limits of > 70% sensitivity and >80% specificity for the ClearLLab testing system. All markers met the manufacturer’s specifications and claims, specifically those not typically included in first-line LDT testing, including CD16, CD38, CD56, CD64, CD123, CD200 and TCRγδ. Cases tested and reviewed using ClearLLab 10C were shown to have essentially similar immunophenotypes observed with the use of the LDT, leading to the same overall diagnostic outcome. In certain categories, however, diagnostic specificity matched diagnostic outcomes exactly, that is, there was 100% concordance of measured markers. Such examples include CD19/CD5 dual positivity for diagnosis of chronic lymphocytic leukaemia or CD19/CD10+ dual positivity for a common acute lymphoblastic leukaemia. Although overall diagnostic concordance was 100%, the four compact ClearLLab panels offered timely simultaneous, collated, and concise interpretation for both tumour and normal background populations. This improved efficiency was difficult to achieve, during interpretation and analysis of the predicate LDT, where limited 2–4-colour marker combinations had to be interpreted across in at least 10–12, but often as many as 20, separate panels.

### Comparing ClearLLab 10C results with clinicopathological outcomes

**FIGURE 3:**
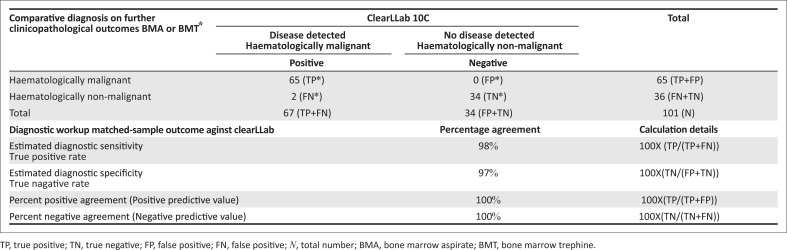
Part 2: Contingency table^34^ comparing the ClearLLab 10C test method (as an objective standard) versus clinicopathological outcomes reported at the Charlotte Maxeke Johannesburg Academic Laboratory in Johannesburg, South Africa, August 2019 – November 2019.

The overall diagnostic sensitivity and specificity were comparable for the outcomes reported on corresponding clinicopathological bone marrow aspirate or trephine findings (Figure 3). The ClearLLab 10C system reliably identified and excluded all diseases and, in two cases, detected the presence of a B-cell lymphoproliferative neoplasm that was not detected morphologically on the corresponding bone marrow aspirate.

## Discussion

This study aimed to verify the performance of a new commercially available, fixed pre-titrated multicolour flow cytometric panel system, the ClearLLab 10C, against an existing LDT that required multiple manual tube setups (often exceeding 10–12 tubes per patient) and required manual titration and separate addition of reagents from a large repertoire of liquid monoclonal antibodies. This study has revealed that the ClearLLab 10C system reliably enables full discovery of most leukaemias and lymphomas, with full concordance to our existing in-house LDT panels. Excellent concordance was also reported against corresponding clinical-pathological outcomes in a subsequent analysis that looked at ClearLLab 10C outcomes against reported morphological findings.

It was expected, however, that the ClearLLab 10C system would have broadly similar clinical outcomes to that generated by the LDT, as both systems used flow cytometry and the same markers to derive outcomes. The multicolour capability of the ClearLLab system versus 2–4-colour panel LDT is what sets the two methods apart; compact access to 27 markers in just four panels, with trackable markers between analyses, is a game-changer, certainly for our site, and a notable improvement over our previous approach to first-line immunophenotypic workup. Specifically, the concise multi-parameter format of the M1, M2, B and T-cell tubes, especially with additional markers that were not included in a typical first-line investigation in our laboratory LDT, including CD16, CD38, CD56, CD64, CD200, CD123 and TCRγδ, has enabled more precise and detailed immunophenotypic insights. Myelomonocytic markers in the M1 tube (CD11b, CD14, CD16, CD64 and HLA-DR) or myeloid markers (CD13, CD15, CD33, CD117 and HLA-Dr) in the M2 tube proved to be a distinct advantage over the LDT to identify and distinguish monocyte from granulocyte lineage, discern all stages of maturation, and identify asynchronous myelomonocytic maturation, including characterising immature forms and blasts. The combination of CD11b, CD13, CD14 and CD16, notably with CD64, facilitated a clear distinction of monocytic maturation and monocyte subsets while also providing insights into granulocytic differentiation. The inclusion of CD16 was especially useful to define both early and late maturation of granulocytes; clustered bright CD16, CD11b and CD13 associated with weak CD10 expression allowed for ease of identification of neutrophils which was helpful in discerning haemodilution of a bone marrow sample. The B and T-cell tubes also enabled simultaneous distinction of lymphoid subsets and provided concise immunophenotypic information about normal or aberrant lymphoid maturation stages. Coincident aberrant loss or asynchronous gain of specific markers was easier to discern using the ClearLLab 10-C panels; in contrast to our 2–4-colour LDT, multiple markers included together in a single ClearLLab panel provided clearer evidence of simultaneous antigen losses or gains of markers. The specific combination of CD45, CD19, CD10, CD20, CD34 and CD200 in the B-cell tube, with HLA-Dr, CD38 and CD19 in the M2 tube, usually reserved for specific disease-profiled LDT panels in our previous practice, has also markedly improved our ability to discriminate early normal B-cell precursors from later mature polyclonal B-cells in first-line workup; CD38-bright expressing, CD45 negative plasma cells are also easily recognised upfront. Further, we have found that CD123, along with CD19, CD34, CD38 and HLA-Dr, identifies B-cells in the M2 tube, which is especially valuable to discern and document discordant expression in normal and abnormal precursor B-cells.^35,36^ Concurrent assessment of most T-cell markers in the T-cell tube was valuable for discerning both immature and mature T-cell lymphoproliferative disorders. Natural killer or cytotoxic cells were easily identified with CD7, CD56 or TCRγδ in the T-cell tube and confirmed with expression of CD16 and CD7 in the M1 tube. From a marker reproducibility perspective, and in stark contrast to our LDT method, the primary backbone markers, CD45 and CD34, included as all four tubes with additional secondary backbone markers, namely CD5, CD7, CD10, CD13, CD38 and HLA-Dr, each repeated across at least two tubes, proved to be enormously helpful to track populations between the four ClearLLab analyses. Another distinct advantage was that substantively fewer cells were needed to achieve a relatively extensive workup in a paucicellular sample using ClearLLab. Often during LDT use, our site would be unable to complete full immunophenotypic workup in paucicellular samples, especially in paediatric and bodily fluid samples like cerebrospinal fluid.

Careful consideration was previously given to implementing the established state-of-the-art EuroFlow system^9,20,37^ that is widely used in Europe. However, the complex EuroFlow demands manual assembly of 8-colour marker panels^21,38^, uses a repertoire of monoclonal antibodies and requires multiple titrations and pipetting protocols. These pose a significant barrier to use in our relatively skills-scarce environment, considering the technical effort needed and potential for error during the dispensing of monoclonal reagent encountered in our existing 2–4-colour LDT. Another factor that hindered the implementation of EuroFlow was the unit’s heavy workload. The unit is a 24-h CD4 laboratory processing up to 12 000 samples per month^39,40^; the technical staff managing the CD4 and HIV immunology bench also support the leukaemia bench. Thus, although the staff are competent to run the CD4 services using pre-titrated and pipetted reagents and standardised, automated testing procedures,^40,41^ they are not primarily trained in flow cytometry. Therefore, from a technical perspective, the compact, pre-titrated ClearLLab 10C fixed tube reagents^27,28^ with automated procedures and standardised instrument setup, allows our laboratory to undertake sophisticated multicolour panel testing confidently while still addressing local challenges. Implementation can significantly reduce leukaemia sample preparation time and improve the efficiency and quality of leukaemia testing in our site.

The difficulties faced in our centre are not unique though. Flow cytometry outcomes vary even in far better-resourced sites.^25^ Despite that testing is undertaken by dedicated and trained flow cytometry laboratory personnel or that there is prescribed standardisation of multicolour methods, deviation from standardised protocols for instrument setup and colour compensation,^25,42^ as well as differences in sample preparation, gating strategies and data interpretation, are reported.^25^ The ClearLLab 10C system can enable our centre to overcome such challenges as it is implemented with standardised flow cytometer multicolour instrument setup and daily prescribed quality control, effectively providing predetermined, user-independent operation in the Navios flow cytometers. The capability for standardised Kaluza C™ hierarchical immunophenotypic analysis of four fixed tubes can also harmonise consistent gating approaches and reporting strategies between reporting pathologists, which is especially important for standardised disease interpretation.

Commercial lyophilised panels, like the ClearLLab 10C system evaluated in this study, are also expected to considerably reduce the technical effort and time needed for sample preparation and analysis. For example, the implementation of ClearLLab will drastically reduce sample preparation and data acquisition time for our first-line investigation of patient samples and reduce the number of (variable) LDT monoclonal reagent panels prepared from around 20 (Table 1) to just four standardised panels (see Table 2) with an estimated reduction in sample preparation from 1.5 h to ~30 min. A notable advantage of the ClearLLab tubes is that antibodies are pre-titrated so that no monoclonal reagents will be added; this is in striking contrast to our previous LDT practice, where up to 40 or more individual monoclonals had to be pipetted into multiple tubes, in varying combinations with the potential of multiple errors (see Table 1). Our previous large inventory of liquid-reagent monoclonal antibodies also required considerable staff time to manage procurement and stock-taking. Pre-dispensed reagent obviates the need for time-consuming flow cytometric titration experiments as well.

### Limitations

Firstly, while there was 100% concordance between the LDT and ClearLLab outcomes, only 101 case reviews were included. Secondly, although the ClearLLab instrument auto-setup makes managing daily quality control and review of complicated multicolour analyses much quicker, easier and standardised, the auto-setup itself still requires training of operators. Attending pathologists also need training and are required to familiarise themselves with Kaluza software and patterns of normal and aberrant haematological population maturation. Thirdly, the ClearLLab 10C M1, M2, T and B-cell panels allow for reasonably extensive discovery of most leukaemias, but there are currently no supplementary ‘second-line’ ClearLLab investigative panels available^7,8^ to discern markers needed for full diagnoses; for example, cytoplasmic studies to discern myeloperoxidase or nuclear terminal deoxynucleotidyl transferase (TdT) expression, CD41, CD42b and CD61 for platelet marker investigation, or extended B-cell lymphoproliferative workup, including amongst others CD11c, CD23, CD30, CD43, CD79b and CD123 amongst others, is still performed in our laboratory to enable the World Health Organization classification of haematological disease. Some commercially produced, pre-dispensed, fixed panels marketed for in vitro rare event use^27,38,43,44,45,46,47,48^ could fill the gap. Such products could potentially be modified to extend the diagnostic repertoire of the ClearLLab 10C and provide for multicolour second-line immunophenotypic investigation, especially for B-cell lymphoproliferative disorders and plasma cell dyscrasias, but this needs further study.

### Conclusion

In this study, there was excellent concordance between our LDT and the multicolour BC ClearLLab 10C panels, with 100% sensitivity and specificity recorded against existing LDT methods. There was also excellent concordance of ClearLLab 10C reporting to documented clinicopathological outcomes. The ClearLLab 10C panels, with manufacturer-standardised setup for colour compensation, appropriate quality control and data acquisition on a Navios flow cytometer, provides concise and comprehensive multi-immunophenotyping for typical leukaemias and lymphomas encountered during routine service delivery while substantially simplifying and standardising sample preparation and data acquisition.
